# PLXDC2 enhances invadopodium formation to promote invasion and metastasis of gastric cancer cells via interacting with PTP1B

**DOI:** 10.1007/s10585-022-10168-5

**Published:** 2022-06-04

**Authors:** Bin Wu, Yan-xia Wang, Jun-jie Wang, Dong-fang Xiang, Meng-si Zhang, Ze-xuan Yan, Wen-ying Wang, Jing-ya Miao, Xi Lan, Jia-jia Liu, Zheng-yan Li, Chuan Li, Jun-yan Fan, Jun-yan Liu, Lei Jiang, Sen-lin Xu, You-hong Cui, Feng Qian

**Affiliations:** 1 Department of General Surgery and Center of Minimal Invasive Gastrointestinal Surgery, Southwest Hospital, Army Medical University (Third Military Medical University), No. 30 Gaotanyan Street, Chongqing, 400038 China; 2 Institute of Pathology and Southwest Cancer Center, Southwest Hospital, Army Medical University (Third Military Medical University), No. 30 Gaotanyan Street, Chongqing, 400038 China

**Keywords:** PLXDC2, Gastric cancer, Metastasis, Invadopodia, Cortactin, PTP1B

## Abstract

Plexin-domain containing 2 (PLXDC2) has been reported as an oncoprotein in several human malignancies. However, its expression and roles in gastric cancer remain largely unclear. In this study, we found that PLXDC2 was highly expressed in gastric cancer tissues, and the expression levels were positively correlated with clinicopathological features, but negatively with the patients’ outcome. Cox regression analysis identified PLXDC2 as an independent prognostic indicator for the patients. Knockdown of PLXDC2 markedly suppressed the in vitro invasion and in vivo metastasis of gastric cancer cells, while overexpression of PLXDC2 resulted in opposite effects. Mechanistically, PLXDC2 enhanced the level of phosphorylated Cortactin (p-Cortactin) by physically interacting with protein tyrosine phosphatase 1B (PTP1B), an important dephosphorylase, to prevent its dephosphorylating of p-Cortactin, thereby promoting the formation of invadopodia. Collectively, our results indicate that PLXDC2 contributes to the invasion and metastasis of gastric cancer by inhibiting PTP1B to facilitate the invadopodium formation, and may serve as a potential prognostic biomarker and a therapeutic target for this disease.

**Supplementary Information:**

The online version contains supplementary material available at 10.1007/s10585-022-10168-5.

## Introduction

Gastric cancer (GC) is the fifth most common cancer and leading cause of cancer-related death worldwide [1]. In China, GC is the second most prevalent cancer and the third most common cause of cancer-related death [2]. Despite the advances in early diagnosis and comprehensive treatment strategies, the outcome of patients with advanced GC remains poor, with median survival around 12 months [3, 4]. Invasion and metastasis are the major contributors of GC progression and poor outcome, but the underlying mechanisms remain unclear. Therefore, a better insight into the mechanisms of GC invasion and metastasis should facilitate the development of more effective therapies for the disease.

Plexin domain containing 2 (PLXDC2) is a nidogen/plexin homology transmembrane protein encoded by PLXDC2*,* which was originally isolated in a gene trap screen for defining brain wiring pattern in mice (mouse line KST37) [5]. It has been identified as a related protein of tumor endothelial marker 7 (TEM7), so it is also referred to TEM7-related (TEM7R) [6]. Subsequent researches revealed that PLXDC2 is involved in proliferation and differentiation of neural progenitors during the development of the nervous system [7], and acts as one of the membrane receptors of pigment epithelial-derived factor (PEDF), an endogenous anti-angiogenesis factor [8]. Recently, aberrant expression of PLXDC2 in cancers has been reported. In breast cancer, elevated PLXDC2 expression at mRNA level was associated with lymph node metastasis and disease progression [9]. It was also overexpressed in hepatocellular carcinoma at protein level [10]. In vulvar squamous cell carcinoma, PLXDC2 has been reported as an unfavorable prognostic marker [11]. In addition, PLXDC2 has been identified as a potential maker to distinguish paclitaxel‑resistant from paclitaxel‑sensitive epithelial ovarian cancers [12]. More recently, Guan et al. reported that PLXDC2 was overexpressed in stromal cell-associated M2 macrophages which associated to EMT and the progression of gastric cancer [13]. However, the expression and clinical relevance of PLXDC2 in GC need to be further investigated.

In the present study, we reported that PLXDC2 was highly expressed in GC tissues, and the expression levels were positively associated with the clinicopathological parameters and negatively with the patients’ overall survival. PLXDC2 enhanced invadopodium formation by physically interacting with PTP1B to prevent its dephosphorylating of p-Cortactin, thereby promoting the invasive and metastatic capabilities of GC cells. Our results reveal PLXDC2 as a novel contributor of invadopodium formation in GC invasion and metastasis, and might act as a promising prognostic indicator as well as a potential therapeutic target for GC.

## Materials and methods

### Patients and tissue specimens

Two GC tissue microarrays (HStmA180Su11 and HStmA180Su17) were obtained from Outdo Biotech Co., Ltd. (Shanghai, China), which contained a total of 170 cases with tumor and pared adjacent normal tissues. These tissue microarrays were used to measure the expression levels of PLXDC2 by immunohistochemical staining (IHC). The clinicopathological characteristics of the cases in the tissue microarrays were listed in Supplementary Table S1.

Another 20 pairs of fresh GC specimens with tumor and pared adjacent tissues were used for detection of PLXDC2 expression by qRT-PCR and WB, which were also obtained from Outdo Biotech Co., Ltd. (Shanghai, China). All the tissues were stored at – 80 °C until use. No patient received any chemoradiotherapy before surgery. All the procedures were performed in accordance with the principles of the Helsinki Declaration and approved by National Human Genetic Resources Sharing Service Platform (2005DKA21300).

### Analyses of data from public databases

The expression data of PLXDC2 at mRNA level in The Cancer Genome Atlas (TCGA) and 4 GEO datasets of gastric cancer (GSE29272, GSE66229, GSE84433 and GSE84437) were downloaded by using online tool cBioPortal (https://www.cbioportal.org) and GEO database (http://www.ncbi.nlm.nih.gov/geo/), respectively. The detailed information of GEO datasets was listed in Table S2.To compare the expression levels of PLXDC2 in cancer and adjacent normal tissues of GEO datasets, the data were analyzed by using GEO2R online tool (https://www.ncbi.nlm.nih.gov/geo/geo2r/) with |log_2_FC|> 2 and adjust P value < 0.05 [14].

Differentially expressed genes (DEGs) were selected using R software 3.6.2 version (www.r-project.org, R Foundation for Statistical Computing, Vienna, Austria) and the Bioconductor project package (http://www.bioconductor.org/) [14] with |log_2_FC|> 2 and adjust P value < 0.05. All enrichment analyses were performed using the database for annotation, visualization and integrated discovery (DAVID, http://david.abcc.ncifcrf.gov/) [15].

### Immunohistochemical staining (IHC) and immunoreactivity score

IHC was performed on 4 μm tissue microarrays or xenograft tumor sections according to the manufacturer’s instructions of the Dako REAL EnVision Detection System (Dako, Denmark). After deparaffinized, rehydrated, antigen retrieval and blocking, the slides were incubated with a rabbit anti-PLXDC2 antibody (1:300, Abcam, Cambridge, UK) or a rabbit anti-p-Cortactin antibody (1:50, CST, USA) at 4 °C overnight. After removing the primary antibody with PBS, a horseradish peroxidase (HRP)-conjugated secondary antibody (Dako) was added and incubated at 37 °C for 1 h. All slides were stained by 3, 39-diaminobenzidine (DAB) (Dako, Denmark) and counterstained with Hematoxylin.

The staining signal of PLXDC2 was scored as previously described [16]. Briefly, five random IHC images of each spot for tissue microarrays or each slide for xenograft tumor sections were captured on the photograph scanned by Pannoramic scanner with case viewer, and analyzed by Image-Pro Plus 6.0 software (Media Cybernetics, Inc., Bethesda, MD, USA). The area sum and integrated optical density (IOD) sum were measured in pixels. The expression intensity of PLXDC2 was presented by the mean value of IOD sum/area sum of 5 photographs for each spot or slide. To ensure data comparability, the same parameter settings were kept for all photographs. The best predictive cut-off value of expression intensity was determined to be 0.138 analyzed with SPSS 25.0 (IBM SPSS Inc., USA) (Fig. S1). The cases with expression intensity ≥ 0.138 were defined as PLXDC2^high^, otherwise were defined as PLXDC2^low^.

### Cell transfection

Lentivirus particles containing short hairpin RNAs targeting PLXDC2 (sh-PLXDC2) and scramble (Mock) were designed, synthesized and packaged by GenePharma (Shanghai, China). The sequences of sh-PLXDC2 and scramble were listed in Table S3. For PLXDC2 and PTP1B overexpression, lentiviral particles containing human full-length PLXDC2 (with c-Myc tag) and PTP1B were constructed and packaged by Shanghai SunBio Biomedical technology (Shanghai, China). All lentivirus particles were used to infect GC cells with 2 μg/mL polybrene, and stably transfected cells were selected by 3 μg/mL puromycin (Sigma-Aldrich, USA). The efficacy of knockdown or overexpression was evaluated by qRT-PCR and WB at mRNA and protein levels, respectively.

The sequences of siRNAs targeting Cortactin, PTP1B and scrambles were designed and synthesized by GeneChem (Shanghai, China) (Table S4). Cell transfection was performed using LipofectamineTM 3000 reagent (Invitrogen, USA) according to the manufacturer’s instructions.

### Wound healing and Metrigel-transwell invasion assays

Wound healing assay was performed as previously described [17]. Briefly, 10 μg/mL mitomycin C (Sigma Chemical, USA) were used for 2 h to inhibit proliferation. Cells were wounded straightly using 10 μL sterile pipette tips, washed twice with PBS gently and then incubated in serum-free RPMI-1640 medium. The wound closures were visualized at 0 and 24 h using an Olympus microscope (Olympus IX50; Olympus, Tokyo, Japan). The wound healing rate (%) was presented as the percentage of the recovered gap distance compared with the initial wound width.

Matrigel-transwell invasion assay was performed as previously described [18]. Briefly, 8.0 μm pore size Transwell inserts (Corning, USA) were precoated with Matrigel (Matrigel: serum-free RPMI-1640 medium = 1:2) (BD, USA). Cells were suspended in serum-free RPMI-1640 and added into the upper chambers (4 × 10^4^ cells in 200 μL per well). The lower chambers were filled with 500 μL RPMI-1640 medium containing 10% FBS. Following 18 h of incubation, invaded cells on the lower surface of the membranes were stained and counted under a microscope from five random fields.

### Quantitative real-time PCR (qRT-PCR)

qRT-PCR analysis was performed as previously described [17]. The primers used in this study were listed in Table S5. The relative mRNA levels were normalized against β-actin using the 2 − ΔΔCt formula.

### Western blotting

Western blotting analyses were performed as previously described [18]. The primary antibodies used in this study were listed in Table S6. β-actin was used as a loading control.

### Co-immunoprecipitation assay

Co-immunoprecipitation (Co-IP) was performed using a Thermo Scientific Pierce Co-IP kit (Thermo Scientific, USA) following the manufacturer’s protocol. Briefly, 10 μg of each antibody and matched IgG were immobilized on AminoLink Plus coupling resin for 6 h, respectively. After washing with IP Lysis/Wash Buffer and incubation with 500 μL GC cell lysate overnight at 4 °C, the resin was washed again and eluted using Elution Buffer. The eluted proteins were separated by SDS–PAGE and immunoblotted with indicated antibodies as Western blotting analysis.

### Immunofluorescence analysis

For immunofluorescence analysis of cells, the cells were seeded on cover slides coated with 0.01% gelatin in 24-well plates in RPMI-1640 medium supplemented with 10% FBS and grown to 50% confluence and then fixed in 4% paraformaldehyde for 30 min at room temperature. For immunofluorescence analysis of tissue slides, the GC tissues were first fixed with Tissue-Tek O.C.T. Compound (SAKURA, USA) and prepared into 4 μm slide. Both cell cover slides and tissue slides were permeabilized with 0.5% Triton X-100 for 30 min. After blocked with 10% of goat blocking serum (ZSGB-Bio, ZLI-9022) for 30 min, slides were incubated with first antibodies, including a rabbit anti-PLXDC2 antibody (1:200, Abcam, Cambridge, UK), anti-MMP14 and a rabbit anti-PTP1B antibody (1:100, Proteintech, China) at 4 °C overnight. After removing the primary antibody with PBS, cell cover slides and tissue slides were incubated with fluorophore-conjugated secondary antibody for 1 h. F-actin were stained with Alexa Fluor® 555 Phalloidin (1:20, CST, USA) for 1 h. Cell nuclei were counterstained with the 4,6-diamidino-2-phenylindole (DAPI). Images were obtained using an inverted confocal laser microscopy (LSM900, Zeiss, Germany).

Invadepodia were defined as the yellow spot of MMP14, a marker of invadopodium [19], co-locating with F-actin.

The percentage of cells with invadopodia was calculated from 5 random fields (10× about 50 cells per field) under the laser confocal microscope.

The number of invadopodia in invadopodium-positive cells was quantified and expressed as the Pearson’s coefficient of co-localization (MMP14 and F-actin) analyzed with JACoP plugin of Image-Pro Plus 6.0 software (Media Cybernetics, Inc., Bethesda, MD, USA) from 5 random fields (100×) [20].

### Intraperitoneal metastasis assay

Female BALB/c nude mice (5 weeks old and 17–20 g weight) were obtained from Byrness Weil biotech Ltd (Chongqing, China) and housed in a pathogen-free environment. The mice were randomly divided into four groups of mock/sh-PLXDC2 and Ctrl/OE-PLXDC2, with six mice in each group. Accordingly treated GC cells were suspended in PBS and injected intraperitoneally at 2 × 10^5^ cells in 200 μL per mouse. The mice were sacrificed at the end of 5th week after the implantations, and numbers of intraperitoneal tumor nodules were photographed and counted. All animal procedures were approved by Laboratory Animal Welfare and Ethics Committee of Third Military Medical University (Army Medical University) (AMUWEC20201544).

### Statistics

All experiments were performed at least three times and Statistical data were presented as the mean ± standard deviation using SPSS or GraphPad Prism software. Comparisons between two groups were analyzed by Student’s *t* test, and One-way ANOVA was used to compare data containing more than two groups. Survival analysis was performed using the Kaplan–Meier method, and survival rates were compared using the log-rank test. Pearson χ^2^ test were used to evaluate the relationship between PLXDC2 expression and clinicopathological features in patients with GC. Hazard ratios (HRs) and 95% confidence intervals (CIs) were used to estimate the correlation between PLXDC2 expression and OS. Cox proportional hazards regression was used for univariate and multivariate survival analyses. Multivariate analysis was performed to identify independent prognostic factors among significantly correlated variables. *P* < 0.05 was considered statistically significant.

## Results

### PLXDC2 is highly expressed in gastric cancer tissues and correlated with clinicopathological features of the patients

**Fig. 1:**
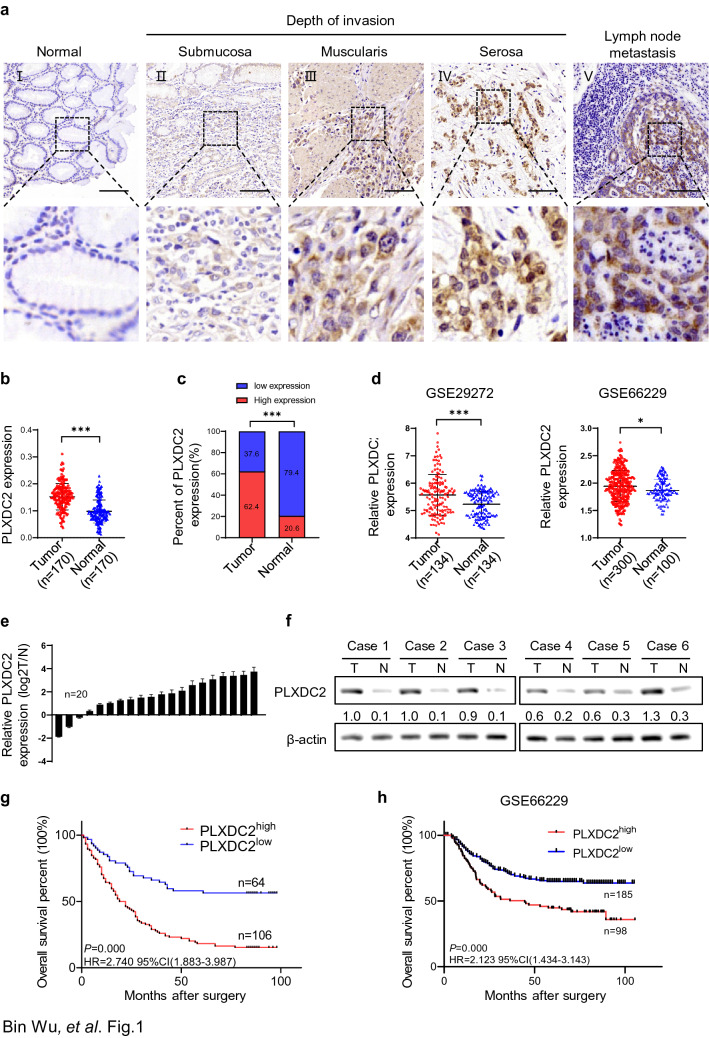
PLXDC2 is highly expressed in gastric cancer tissues and associated with poor outcome of the patients. **a** Representative immunohistochemical staining (IHC) images of PLXDC2 expression in adjacent normal tissue, gastric cancer tissues with different invasion depth and metastatic focus. Scale bar = 50 μm. **b** The IHC scores of PLXDC2 expression in GC tumor tissues were significantly higher than that in adjacent normal tissues. ***, *P* < 0.001. **c** High expression of PLXDC2 was more frequent in GC tumor tissues than in adjacent normal tissues (Pearson *χ*^2^ test). ***, *P* < 0.001. **d** mRNA level of PLXDC2 expression in GEO GSE29272 and GSE66229 datasets was higher in tumor tissues than in adjacent normal tissues. ***, *P* < 0.001, *, *P* < 0.05. **e** mRNA levels of PLXDC2 expression were higher in 20 fresh surgical GC tumor tissues than in paired adjacent normal tissues. **f.** Protein levels of PLXDC2 expression were higher in six fresh surgical GC tumor tissues (T) than in adjacent normal tissues (N). **g** Kaplan–Meier estimation indicated that the overall survival rates of patients with PLXDC2^high^ were significantly lower than that with PLXDC2^low^ patients (*P* = 0.000, HR = 2.740, 95%CI (1.883–3.987)). **h** Analyses on the data in GEO GSE66229 showed that the overall survival rates of patients with PLXDC2^high^ were significantly lower than that with PLXDC2^low^ patients (*P* = 0.000, HR = 2.123, 95%CI (1.434–3.143))

The expression of PLXDC2 protein in 170 GC specimens with tumor and adjacent normal tissues was examined by IHC. PLXDC2 staining was low or absence in adjacent normal tissues, but high in tumor tissues and metastatic lymph node, mainly in cytoplasm and membrane of GC cells (Fig. 1a_I–V_, b). Moreover, the intensity of PLXDC2 staining in tumor tissues was increased with invasion depth (Fig. 1a_II–IV_). The proportion of high expression of PLXDC2 (PLXDC2^high^) defined by the optimal cutoff value of 0.138 (Fig. S1) was significantly higher in tumor tissues than in adjacent normal tissues (62.4%, 106/170 *vs.* 20.6%, 35/170; *P* = 0.001) (Fig. 1c). These results were supported by analyses in GEO datasets, including GSE29272 (n = 134, *P* = 0.000) and GSE66229 (n = 400, *P* = 0.016) (Fig. 1d). To confirm the results of IHC, we measured the expression of PLXDC2 at mRNA and protein levels in 20 and 6 pairs of fresh GC tumor and adjacent tissues by qRT-PCR and Western blotting, respectively. Both at mRNA and protein levels, the expressions of PLXDC2 were significantly elevated in tumor tissues as compared to adjacent normal counterparts (Fig. 1e, f).

We then investigated the clinical relevance of PLXDC2 expression in GC. The analyses showed that upregulation of PLXDC2 was positively correlated with Neoplasm Histological Grade (*P* = 0.000), TNM Stage (*P* = 0.000), T Stage (*P* = 0.007), N Stage (*P* = 0.008), but not with Age (*P* = 0.205), Sex (*P* = 0.147), Tumor Size (*P* = 0.916) and Tumor Location (*P* = 0.369) (Table 1). Kaplan–Meier survival analysis showed that patients with PLXDC2^high^ GC had a shorter overall survival (OS) compared to those with PLXDC2^low^ tumors (*P* = 0.000, HR = 2.740 95% CI (1.883–3.987)) (Fig. 1g). Both univariate and multivariate Cox regression analyses revealed that PLXDC2 expression was an independent prognostic factor for OS in GC patients (*P* = 0.000 and 0.007, respectively) (Table 2). To confirm these results, we further analyzed the data in GEO dataset GSE66229. Kaplan–Meier survival analysis declared that PLXDC2^high^ patients had a significantly lower overall survival rate as compared to PLXDC2^low^ patients (*P* = 0.000 HR = 2.123 95% CI (1.434–3.143)) (Fig. 1h). These results demonstrate that PLXDC2 is a novel pro-malignant factor in GC.

**Table 1:** The relationship between PLXDC2 expression and clinicopathological features of GC patients

Clinicopathological features	Number	PLXDC2 expression		χ^2^	*P* value
		High (%)	Low (%)		
Age (years)				1.604	0.205
< 68^a^	85	49(57.6)	36(42.4)		
≥ 68	85	57(67.1)	28(32.9)		
Sex				2.105	0.147
Male	119	70(58.8)	49(41.2)		
Female	51	36(70.6)	15(29.4)		
Neoplasm histological grade				13.569	**0.000**
G1 + G2	70	32(45.6)	38(54.4)		
G3	110	83(73.5)	27(26.5)		
TNM stage				13.923	**0.000**
I + II	68	31(47.1)	37(52.9)		
III + IV	102	75(74.5)	27(25.5)		
T stage				9.939	**0.007**
T1 + T2	20	9(45)	11(55)		
T3	114	67(58.8)	47(41.2)		
T4	36	30(83.3)	6(16.7)		
N stage				11.791	**0.008**
N0	45	20(44.4)	25(55.6)		
N1	23	12(52.2)	11(47.8)		
N2	43	32(74.4)	11(25.6)		
N3	59	42(72.6)	17(27.4)		
Tumor size (cm)				0.011	0.916
< 5	115	72(62.6)	43(37.4)		
≥ 5	52	33(63.5)	29(36.5)		
Tumor location				1.993	0.369
Proximal gastric	25	16(64.0)	9(36.0)		
Middle gastric	58	40(69.0)	18(31.0)		
Distal gastric	87	50(57.5)	37(42.5)		

*SRP* surveillance research program in *NCI'sDCCPS* NCI's division of cancer control and population sciences

^a^Stomach cancer is most frequently diagnosed among people aged 65-74, and the median age at diagnosis is 68 (Surveillance Research Program (SRP) in NCI's Division of Cancer Control and Population Sciences (DCCPS))

Statistically significant *P* values are in bold

**Table 2:** Univariate and multivariate Cox regression analyses of overall survival in patients with gastric cancer

Prognostic variables	Univariate analysis		Multivariate analysis	
	*P* value	HR (95% CI)	*P* value	HR (95% CI)
PLXDC2 expression	**0.000**	13,560.686 (412.328–445,985.088)	**0.007**	191.715(4.069–9033.841)
Age (years)	0.056	1.017 (0.999–1.036)	–	–
Sex	0.216	1.279(0.871–1.878)	–	–
Neoplasm histological grade	**0.027**	1.516(1.042–2.206)	–	–
TNM stage	**0.000**	3.046(2.087–4.443)	–	–
T stage	**0.000**	1.787(1.329–2.402)	**0.012**	1.533(1.099–2.138)
N stage	**0.000**	4.728(2.692–8.305)	**0.000**	3.882(2.193–6.871)
Tumor size	**0.000**	1.103(1.058–1.149)	**0.001**	1.081(1.032–1.132)
Tumor location	**0.018**	1.273(1.043–1.555)	–	–

Statistically significant *P* values are in bold

### PLXDC2 promotes in vitro invasion and in vivo metastasis of GC cells

**Fig. 2:**
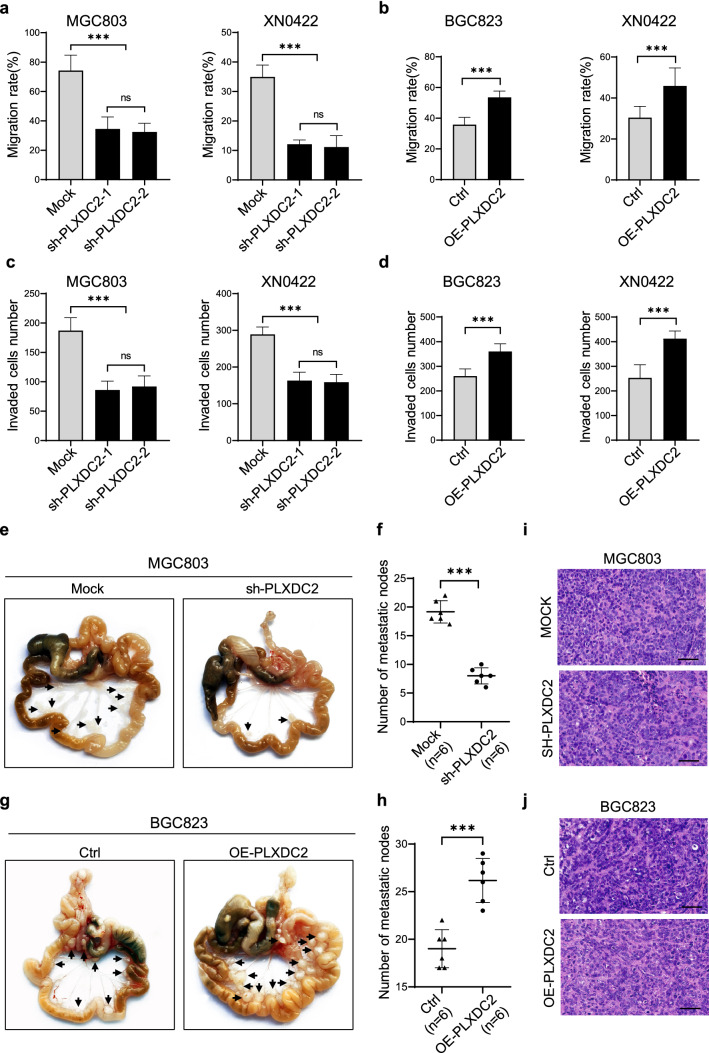
PLXDC2 promotes migration and invasion of gastric cancer cells in vitro and metastasis in vivo. **a** Quantification of wound healing assays showed decreased migration ability in PLXDC2-knockdown MGC803 and XN0422 cells as compared with their Mock cells. ***, *P* < 0.001, ns, no significance. **b** Quantification of wound healing assays showed increased migration ability of PLXDC2 overexpression BGC823 and XN0422 cells as compared with their control (Ctrl) cells. ***, *P* < 0.001. **c** Quantification of Matrigel-transwell invasion assays showed decreased invasive ability of PLXDC2-knockdown MGC803 and XN0422 cells as compared with their Mock cells. ***, *P* < 0.001, ns, no significance. **d** Quantification of Matrigel-transwell invasion assay showed increased invasive ability in OE-PLXDC2 BGC823 and XN0422 cells as compared with their Ctrl cells. ***, *P* < 0.001. **e** Representative images of intraperitoneal metastasis model showed that sh-PLXDC2 cells formed less metastatic nodules than Mock cells. Black arrows indicate intraperitoneal nodules. **f** Quantification of peritoneal metastatic foci derived from PLXDC2-knockdown and Mock cells. n = 6, ***, *P* < 0.001. **g** Representative images of intraperitoneal metastasis model showed that OE-PLXDC2 cells formed more metastatic nodules than Ctrl cells. Black arrows indicate intraperitoneal nodules. **h** Quantification of peritoneal metastatic foci derived from PLXDC2-overexpression and Ctrl cells. n = 6, ***, *P* < 0.001. **i** and **j** Representative H&E images confirmed the GC origin of metastatic foci derived from both PLXDC2 knockdown (**i**) and overexpression (**j**) cells. Scale bar = 50 μm

The above clinical findings suggested the involvement of PLXDC2 in invasion and metastasis of GC. To address this issue, we first established the GC cell models with PLXDC2-knockdown and -overexpression. Among four GC cell lines (BGC823, MGC803, SGC7901 and XN0422) and an immortalized gastric epithelium cell line GES-1, GC cell lines showed higher PLXDC2 expression than GES-1(Fig. S2a and b). GC cell lines also exhibited different expression levels of PLXDC2, with the highest in MGC803, the lowest in BGC823, and the middle in XN0422, so they were used to establish PLXDC2-knockdown (sh-PLXDC2), PLXDC2-overexpression (OE-PLXDC2), and sh-PLXDC2/ OE-PLXDC2 cell models, respectively (Fig. S3). Compared with Mock cells, sh-PLXDC2-MGC803 and -XN0422 cells showed significantly decreased wound healing capabilities (Fig. 2a and Fig. S4a), whereas the wound healing capabilities of OE-PLXDC2-BGC823 and -XN0422 cells were markedly enhanced as compared to control cells (Fig. 2b and Fig. S4b). Identically, the cell invasion capacities were attenuated by PLXDC2 knockdown in MGC803 and XN0422 but enhanced by PLXDC2 overexpression in BGC823 and XN0422 (Fig. 2c, d and Fig. S4c, S4d). The intraperitoneal metastasis models showed that the mice implanted with sh-PLXDC2-MGC803 cells generated fewer metastatic nodules than those implanted with Mock cells (*P* = 0.000) (Fig. 2e, f), whereas mice implanted with OE-PLXDC2 BGC823 cells formed more metastatic foci than controls (*P* = 0.000) (Fig. 2g, h). H&E staining confirmed that the metastatic nodules were derived from GC cells (Fig. 2i, j). These results indicate that PLXDC2 is closely involved in the invasion and metastasis of GC cells.

### Analyses of RNA-Seq and public database to predict the potential mechanism of PLXDC2 enhancing invasion and metastasis in GC cells

**Fig. 3:**
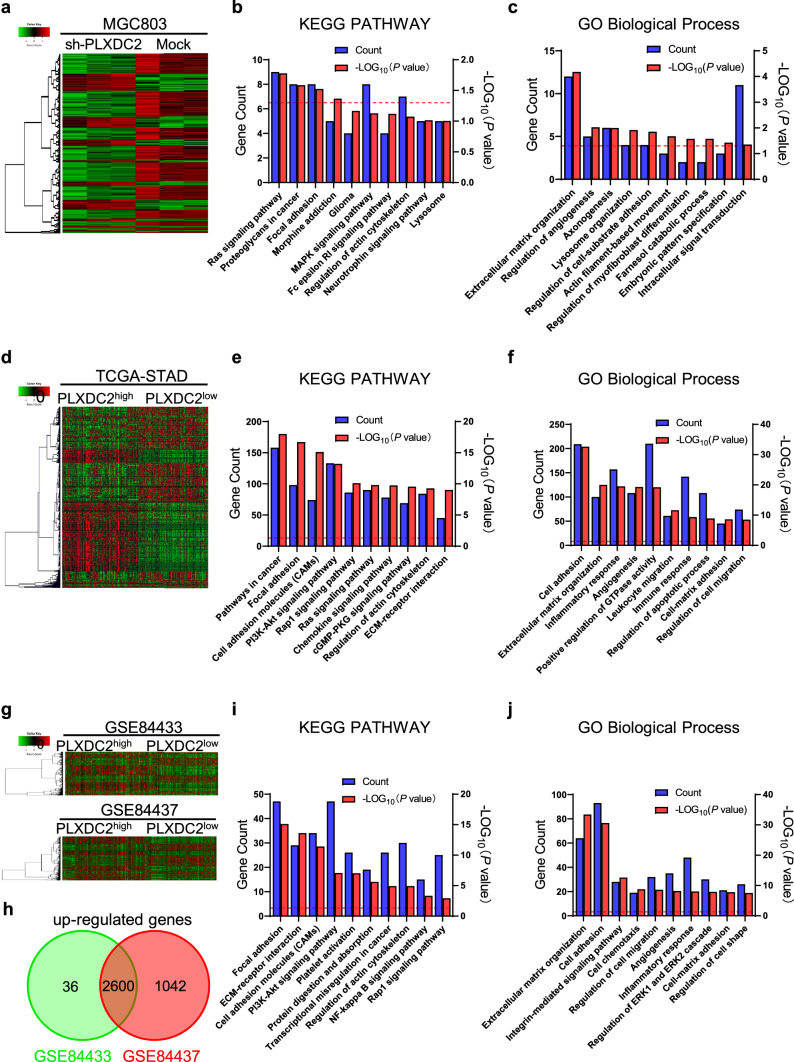
Data of RNA-Seq and public database predict the potential mechanism of PLXDC2-enhancing invasion and metastasis. **a** The heatmap of RNA-Seq with sh-PLXDC2 and mock MGC803 cells. **b** Top 10 of KEGG pathway enrichment terms in down-regulated differently expressed genes (DEGs). Blue bar, gene count; red column, -Log_10_(*P* value); red dotted line indicates -Log_10_(*P* value) = 1.3 (*P* = 0.05). **c** Top 10 of GO biological processes enrichment terms in down-regulated DEGs. GO, Gene ontology. Blue bar, gene count; red column, -Log_10_(*P* value); red dotted line indicates -Log_10_(*P* value) = 1.3 (*P* = 0.05). **d** RNA-Seq heatmap of DEGs between PLXDC2^high^ and PLXDC2^low^ cases in TCGA database. **e** Top 10 of KEGG pathway enrichment terms in up-regulated DEGs in TCGA database. Blue bar, gene count; red column, -Log_10_(*P* value); red dotted line indicates -Log_10_(*P* value) = 1.3 (*P* = 0.05). **f** Top 10 of GO biological processes enrichment terms in up-regulated DEGs in TCGA database. Blue bar, gene count; red column, -Log_10_(*P* value); red dotted line indicates -Log_10_(*P* value) = 1.3 (*P* = 0.05). **g** Microarray heatmap of DGEs between PLXDC2high and PLXDC2low cases in GEO GSE84433 and GSE84437 datasets. **h** Venn diagram identified consensus 2600 DEGs upregulated in GSE84433 and GSE84433 datasets. **i** Top 10 of KEGG pathway enrichment terms of the up-regulated DEGs. Blue bar, gene count; red column, -Log_10_(*P* value); red dotted line indicates -Log_10_(*P* value) = 1.3 (*P* = 0.05). **j** Top 10 of GO biological processes enrichment terms of the up-regulated DEGs. Blue bar, gene count; red column, -Log_10_(*P* value); red dotted line indicates -Log_10_(*P* value) = 1.3 (*P* = 0.05). (Color figure online)

To explore the mechanisms of PLXDC2 enhancing invasion and metastasis in GC cells, we performed an RNA-Seq analysis with sh-PLXDC2 MGC803 and Mock cells (Fig. 3a). A total of 264 down-regulated differentially expressed genes (DEGs) were identified (|Expr Fold Change|≥ 1.5, *P*-value < 0.05). KEGG PATHWAY enrichment analysis with these down-regulated DEGs showed that focal adhesion is one of the most enriched pathways after knockdown of PLXDC2 (*P* = 0.030) (Fig. 3b). Biological process (BP) analysis of GO showed that extracellular matrix organization, regulation of cell-substrate adhesion, and actin filament-based movement were enriched (*P* = 0.000, *P* = 0.014 and *P* = 0.021, respectively) (Fig. 3c). To confirm these results, we further analyzed the data of TCGA database and GSE84433 and GSE84437 datasets. The patients in TCGA-STAD cohort and GEO datasets were divided into PLXDC2^high^ and PLXDC2^low^ groups based on cutoff value and median, respectively. In TCGA-STAD cohort, there were 4233 upregulated DEGs (log2 Fold change ≥ 2 and *P*-value < 0.05) in PLXDC2^high^ group as compared with PLXDC2^low^ group (Fig. 3d). KEGG analysis showed focal adhesion, cell adhesion molecules and regulation of actin cytoskeleton were also enriched (Fig. 3e). BP analysis of GO showed that cell adhesion, extracellular matrix organization, cell–matrix adhesion and regulation of cell migration were enriched (Fig. 3f). In GSE84437 and GSE84433 datasets, 2600 consensus up-regulated DGEs were identified (Fig. 3g, h). Focal adhesion, cell adhesion molecules, and regulation of actin cytoskeleton were enriched in KEGG analysis (Fig. 3i), and extracellular matrix organization, cell adhesion, regulation of cell migration and cell–matrix adhesion were enriched in BP analysis (Fig. 3j). All the enrichment analyses indicate that PLXDC2 is involved in focal adhesion, extracellular matrix organization and actin cytoskeleton regulation in GC cells, implying that the cell motility-associated structures are regulated by PLXDC2 in GC cells.

### PLXDC2 modulates invadopodium formation to facilitate the invasion of GC cells

**Fig. 4:**
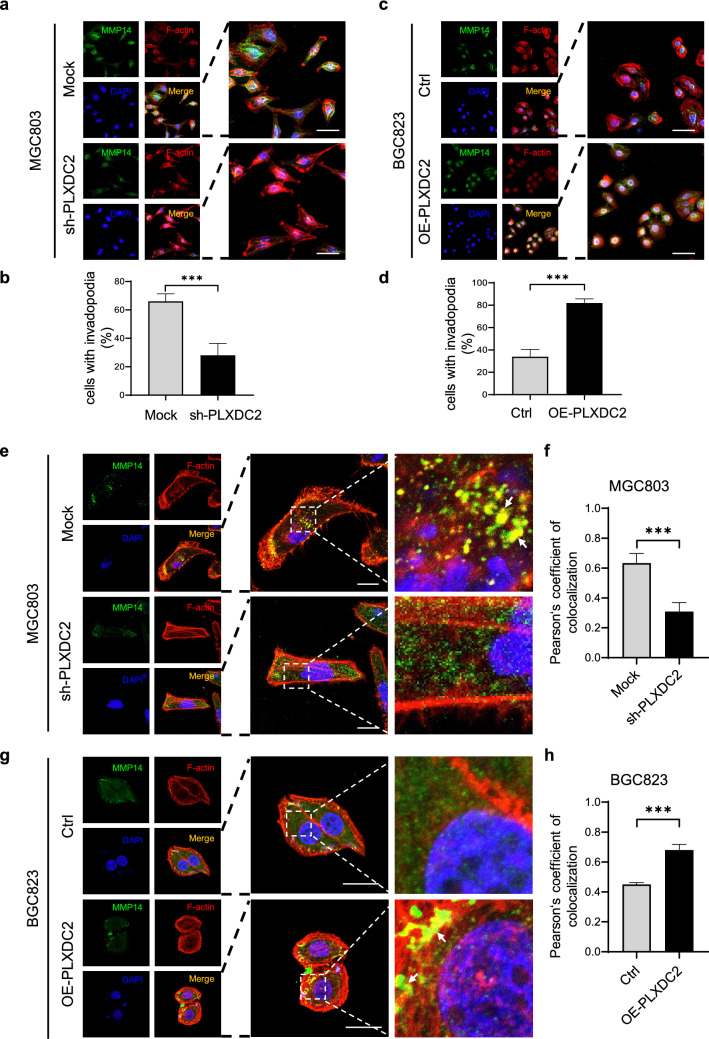
PLXDC2 is involved in invadopodium formation in GC cells. **a** Representative IFC images showed that PLXDC2 knockdown decreased the proportion of invadopodia positive MGC803 cells. Invadopodia were defined as the co-localization (yellow spots) of MMP14 (green), an invadopodium marker, and F-actin (red). DAPI staining showed the Nuclei (blue). Scale bar = 40 μm. **b** Statistical histogram showed reduced percentage of invadopodium-positive cells in PLXDC2-knockdown MGC803 cells, as compared to Mock cells (5 random fields (10 ×), about 50 cells/field). ***, *P* < 0.001. **c** Representative IFC images showed that PLXDC2 overexpression increased proportion of invadopodia positive BGC823 cells. **d** Statistical histogram showed that PLXDC2 overexpression increased the percentage of BGC823 cells with invadopodia. ***, *P* < 0.001. **e** Representative IFC images showed that PLXDC2 knockdown decreased the number of invadopodia in indopodium positive MGC803 cells. White arrows indicate invadopodia. **f** Statistical histogram showed that PLXDC2 knockdown decreased number of invadopodia in indopodium positive MGC803 cells, which was expressed as Pearson’s coefficient of co-localization (MMP14 and F-actin) from 5 random fields (100 ×). ***, *P* < 0.001. **g** PLXDC2 overexpression increased number of invadopodia in indopodium positive BGC823 cells. White arrows indicate invadopodia. **h** Statistical histogram showed that PLXDC2 overexpression increased the number of invadopodia in indopodium positive BGC823 cells (100 × , 5 random fields). ***, *P* < 0.001. (Color figure online)

The cell motility-associated structures formed in invading cancer cells are protrusive structures and are driven by actin cytoskeleton reorganization [21, 22]. The protrusive structures mainly include lamellipodia, filopodia and invadopodia, and the invadopodium is considered to be a hallmark of tumor cells that undergo systemic dissemination and metastasis [23]. Therefore, we further clarified whether PLXDC2 was involved in invadopodium formation in GC cells by employing Matrix metalloproteinase 14 (MMP14; also known as Membrane-type 1 matrix metalloproteinase, MT1-MMP) as the marker of invadopodia [24] and used an immunofluorescence cytochemical staining that defined invadopodia as a co-localization of MMP14 and F-actin[19, 25]. PLXDC2 knockdown markedly reduced the percentage of invadopodium-positive cells in MGC803 and XN0422 cell lines, while PLXDC2 overexpression significantly increased the percentage of invadopodium-positive cells in BGC823 and XN0422 cell lines (Fig. 4a–d and Fig. S5a-d). Moreover, the number of invadopodium formation in invadopodium-positive cells was obviously decreased in PLXDC2-knockdown MGC803 and XN0422 cell lines, but markedly increased in PLXDC2-overexpressin BGC823 and XN0422 cell lines (Fig. 4e–h and Fig. S5 e–h). These results indicate that PLXDC2 is an important molecule promoting invadopodium formation in GC cells.

### Phosphorylated Cortactin mediates PLXDC2-promoted invadopodium formation

**Fig. 5:**
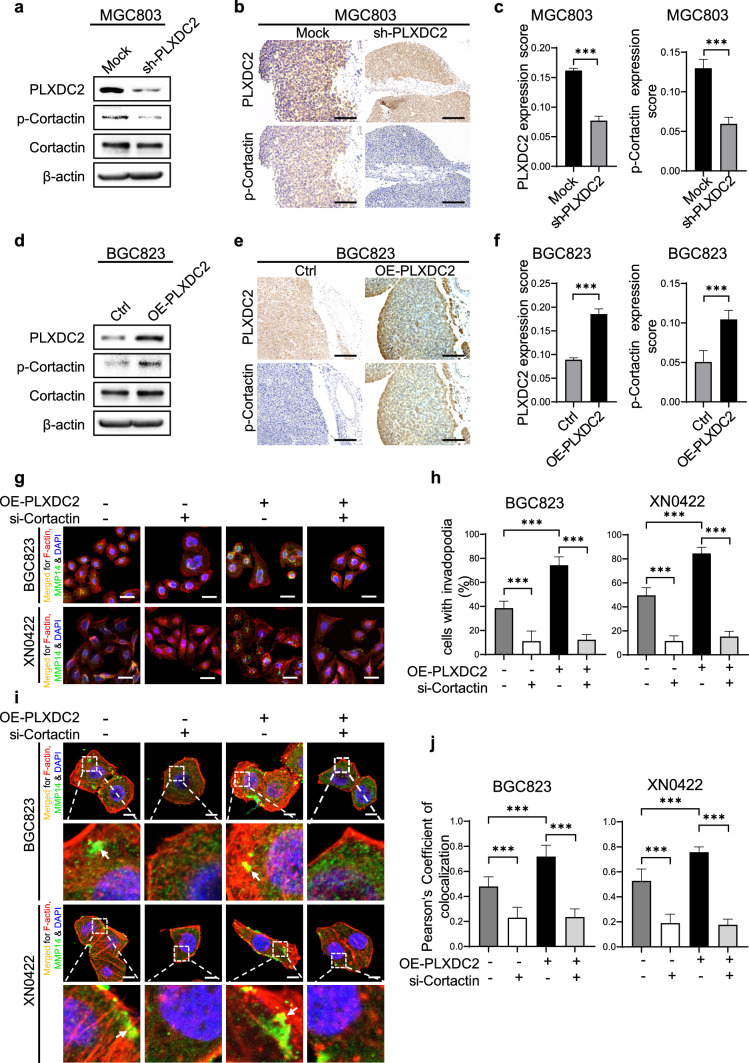
Phosphorylated Cortactin mediates PLXDC2-induced invadopodium formation. **a** Western blotting analysis showed that PLXDC2 knockdown significantly reduced the level of phosphorylated Cortactin (p-Cortactin) in MGC803 cells. **b** Representative IHC images showed that the expression level of PLXDC2 in metastatic foci of mouse peritoneal metastasis model derived from PLXDC2-knockdown MGC803 cells was lower than that derived from Mock cells (upper panel). The expression levels of p-Cortactin were changed consistently with PLXDC2 expression (lower panel). **c** Statistical histograms of IHC scores for PLXDC2 and p-Cortactin expression in metastatic foci of mouse peritoneal metastasis model derived from PLXDC2-knockdown and Mock MGC803 cells. n = 6 for each group. ***, *P* < 0.001. **d** Western blotting analysis showed that PLXDC2 overexpression significantly increased the level of p-Cortactin in BGC823 cells. **e** Representative IHC images showed that PLXDC2 expression in metastatic foci of mouse peritoneal metastasis model derived from PLXDC2 overexpression BGC823 cells was higher than their Ctrl cells (upper panel). The expression levels of p-Cortactin were changed consistently with PLXDC2 expression (lower panel). **f** Statistical histograms of IHC scores for IHC scores of PLXDC2 and p-Cortactin expression in metastatic foci derived from PLXDC2-overexpression and Ctrl BGC823 cells. n = 6 for each group. ***, *P* < 0.001. **g **Representative merged IFC images showed that silencing Cortactin decreased the proportion of invadopodium-positive cells in PLXDC2-overexpressing and Ctrl BGC823/XN0422 cells. Yellow spots, invadopodia. Scale bar = 40 μm. **h** Statistical histograms of (5 g). Five random fields (10 ×) with about 50 cells/field. ***, *P* < 0.001. **i** Representative merged IFC images showed that silencing Cortactin decreased the number of invadopodia of invadopodium-positive cells in PLXDC2-overexpressing and Ctrl BGC823/XN0422 cells. White arrows indicate invadopodia. **j** Statistical histograms of (5**i**). The number of invadopodia in invadopodium-positive cells was expressed as the Pearson’s coefficient of co-localization (MMP14 and F-actin) from 5 random fields (100 ×). ***, *P* < 0.001

It is well known that cortical actin binding protein (Cortactin) in its phosphorylated form (p-Cortactin), especially at Y421, acts as a key scaffold protein to initiate the formation of invadopodia [26]. To ascertain whether p-Cortactin is involved in invadopodium formation promoted by PLXDC2, we examined the impact of manipulating PLXDC2 on the level of p-Cortactin in GC cells and in metastatic foci of mouse peritoneal metastasis models. As shown in Fig. 5a and S6a, silencing PLXDC2 markedly reduced the level of p-Cortactin in MGC803 and XN0422 cells. Decreased p-Cortactin staining was also observed in the metastatic foci derived from PLXDC2-knockdown MGC803 cells as compared with the control (Fig. 5b, c). On the contrary, overexpressing PLXDC2 significantly elevated the level of p-Cortactin in BGC823 and XN0422 cells (Fig. 5d, S6b). Compared with metastatic foci derived from control cells, metastatic foci derived from PLXDC2-overexpressing BGC823 cells showed increased p-Cortactin expression (Fig. 5e, f). To confirm the involvement of p-Cortactin in PLXDC2-promoted invadopodium formation, we observed the effect of silencing Cortactin with siRNA on the formation of invadopodia in PLXDC2-overexpressing and control GC cells. Silencing Cortactin markedly reduced the percentage of invadopodium-positive cells both in PLXDC2-overexpressing BCG823 and XN0422 cells and their control cells (Fig. 5g, h). Meanwhile, silencing Cortactin also significantly lowered the number of invadopodia in invadopodium-positive cells of PLXDC2-overexpression BGC823 and XN0422 cells and their control cells (Fig. 5i, j). These results suggest that p-Cortactin acts as an important mediator in the invadopodium formation facilitated by PLXDC2.

### PLXDC2 physically interacts with PTP1B to prevent its dephosphorylation of p-Cortactin

**Fig. 6:**
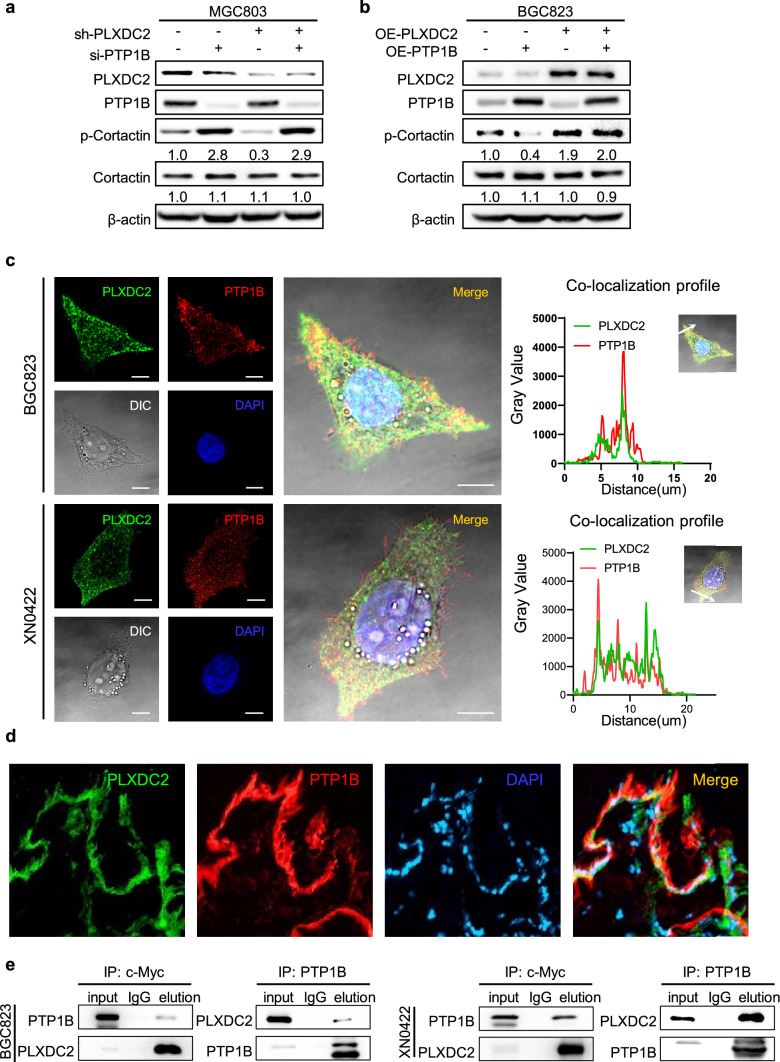
PLXDC2 physically interacts with PTP1B to prevent its dephosphorylating of p-Cortactin. **a** Western blotting analysis showed that knockdown of PLXDC2 in MGC803 cells hardly affected PTP1B expression, but decreased p-Cortactin level. Silencing PTP1B markedly increased the p-Cortactin in of PLXDC2-knockdown Mock MGC803 cells, and reversed the inhibitory effect of PLXDC2 knockdown on p-Cortactin expression in sh-PLXDC2 MGC803 cells. **b** Western blotting analysis showed that overexpression of PLXDC2 in BGC823 cells hardly affected PTP1B expression, but increased p-Cortactin level. Overexpressing PTP1B decreased p-Cortactin in Ctrl BGC823 cells, and hardly reduced the level of p-Cortactin in OE-PLXDC2 BGC823 cells. **c** Representative IFC images of co-localization of PLXDC2 and PTP1B in OE-PLXDC2 BGC823 and XN0422 cells grown on a thin gelatin matrix (Left panel). Scale bar = 10 μm. Right panel showed line tracings representing fluorescence peaks. **d** Representative IFC images showed the co-localization of PLXDC2 and PTP1B in fresh GC tissues. **e** Co-IP showed that PLXDC2 interacted with PTP1B in PLXDC2-overexpressing (with c-Myc tag) BGC823 and XN0422 cells

**Fig. 7:**
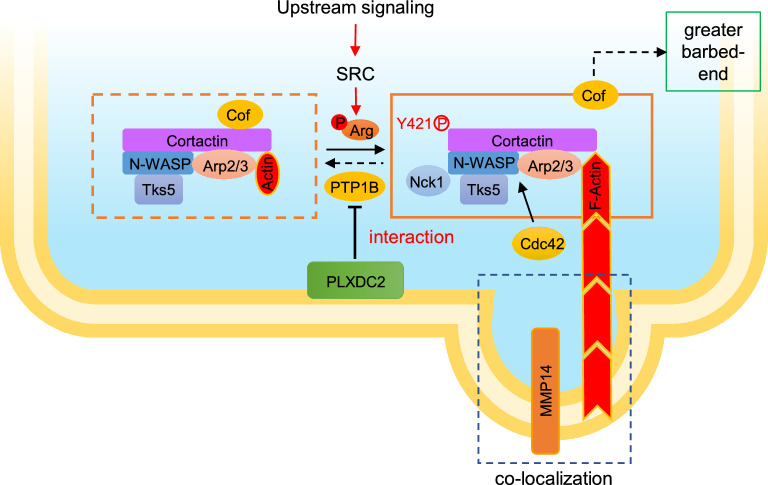
Model of PLXDC2 enhancing formation of invadopodia in GC cells. In the model of invadopodium formation descripted by Eddy et al.[23], phosphorylation of Cortactin at tyrosine 421 is a critical point in invadopodium formation. Tyrosine kinase (mainly Arg) catalyzes the phosphorylation of Cortactin at Y421 in response to its upstream signaling, facilitating the formation of invadopodia. Whereas protein tyrosine phosphatase (mainly PTP1B) catalyzes the dephosphorylation of p-Cortactin at Y421, inhibiting the process of invadopodium formation. PLXDC2 physically interacted with PTP1B to prevent its dephosphorylation of p-Cortactin at Y421, thereby promoting the formation of invadopodium in GC cells

Protein tyrosine phosphatase 1B (PTP1B) has been reported to bind to p-Cortactin and catalyze its dephosphorylation, thereby inhibiting the invadopodium assembly and function in tumor cells [27, 28]. Therefore, we examined whether PTP1B is also involved in PLXDC2-enhanced p-Cortactin in GC cells. Treatment with siRNA targeting PTP1B significantly increased the level of p-Cortactin in sh-PLXDC2 MGC803 mock cells. Compared sh-PLXDC2 MGC803 cells with their Mock cells, knockdown of PLXDC2 hardly affected PTP1B expression, but markedly reduced p-Cortactin level; Silencing PTP1B abolished the inhibitory effect of PLXDC2 knockdown on p-Cortactin expression in sh-PLXDC2 MGC803 cells (Fig. 6a). In OE-PLXDC2 BGC823 control cells, overexpression of PTP1B (Fig. S7) markedly decreased the level of p-Cortactin. Overexpression of PLXDC2 hardly changed the level of PTP1B, but increased p-Cortactin level as compared with its control. The inhibitory effect of overexpressing PTP1B the level of p-Cortactin was abrogated by overexpression of PLXDC2 (Fig. 6b). These results strongly suggest that PTP1B is an important mediator for PLXDC2 to upregulate p-Cortactin in GC cells. Moreover, these results also indicate that PLXDC2 does not affect PTP1B expression but inhibits its function. Hence, we hypothesized that PLXDC2 could interact with PTP1B to prevent its dephosphorylation of p-Cortactin. With immunofluorescence staining, an obvious co-localization of PLXDC2 and PTP1B was observed in OE-PLXDC2 BGC823 and XN0422 cells (Fig. 6c). Consistently, the co-localization of PLXDC2 and PTP1B was also observed in fresh GC tumor tissues (Fig. 6d). The interaction of PLXDC2 and PTP1B was further verified by Co-IP assays in PLXDC2-overexpressing BGC823 and XN0422 cells, where PLXDC2 physically interacted with PTP1B (Fig. 6e). These results suggest that PLXDC2 enhances p-Cortactin level by physically interacting with PTP1B to prevent its dephosphorylation of p-Cortactin. The potential mechanism by which PLXDC2 enhances phosphorylation of Cortactin to promote invadopodium formation is summarized as Fig. 7.

## Discussion

Invasion and metastasis are the important malignant behaviors of GC, but the underlying mechanisms remain to be elucidated. Although the roles of PLXDC2 in cancer have not been fully characterized, several studies have linked PLXDC2 to the metastasis and progression of tumors, such as breast cancer [9] and vulvar squamous cell carcinoma [11]. Moreover, a recent report indicated that PLXDC2 was expressed in stromal cells of gastric cancer and that its crosstalk with tumor-associated macrophages could contribute to cancer biology by inducing the EMT process [13]. In the present study, we demonstrated that PLXDC2 was an important molecule promoting the invasion and metastasis of GC. In addition, we demonstrated that the expression level of PLXDC2 was positively associated with the clinicopathological parameters of GC and negatively associated with the outcome of GC patients.

To successfully metastasize from primary site to distant organs, cancer cells must first penetrate through several physical barriers to escape the primary tumor and entry into the bloodstream, and then spread to distant tissues [29]. These processes rely on cancer cells to form the invadopodium, a specialized F-actin-based membrane protrusion with proteolytic activity that degrades the extracellular matrix (ECM) [23, 30]. Invadopodia are increasingly becoming a hallmark of cancerous cell invasion and metastasis [30], and as a prognostic marker as well as a therapeutic target for cancer metastasis [31]. Up to now, invadopodia have been identified in a number of invasive cancer cells, such as breast cancer [32], glioma [33], pancreatic cancer [34], lung cancer [35], hepatocellular carcinoma [36] and gastric cancer [37]. In the present study, we demonstrated that PLXDC2 facilitated the formation of invadopodia in GC cells, thereby promoting the invasion and metastasis of GC.

The formation of invadopodia is a highly dynamic process, which constitutes a lifecycle containing four successive phases: initiation, assembly, maturation and disassembly [24]. The process of invadopodium formation is also highly complex, involving a large number of proteins and being regulated by many signaling pathways. Cortactin, an actin-binding protein, is closely involved in all the four phases of invadopodium lifecycle [24]. The phosphorylation of two tyrosine residues Y421 and Y466 (in particular Y421) of Cortactin has been shown as a critical point that regulates Cortactin-Nck1 direct interactions and promotes free actin barbed end generation during the process of invadopodium formation [38]. To investigate the potential mechanism of PLXDC2 promoting invadopodium formation, we evaluated the effect of manipulating PLXDC2 expression on Cortactin phosphorylation. Silencing PLXDC2 significantly reduced the level of p-Cortactin at Y421, while overexpressing PLXDC2 resulted in the upregulation of p-Cortactin at Y421, suggesting the involvement of p-Cortactin in the process of PLXDC2 regulating invadopodium formation.

The phosphorylation of Cortactin is regulated by many signaling pathways, in particular the tyrosine kinase pathways c-Src and Arg in response to upstream signaling, such as integrin-adhesion, cadherin-adhesion and growth factor receptors [39, 40]. However, the level of p-Cortactin is also regulated by tyrosine phosphatases. PTP1B has been reported to be an important tyrosine phosphatase involved in invadopodium formation, which is able to physically bind with Cortactin and directly dephosphorylate of p-Cortactin at Y421 and Y446 [27, 28, 41]. Weidmann, et al.reported that Mena^INV^, one of the alternatively spliced isoforms of Mena, is a key regulatory protein to enhance phosphorylation of Cortactin at tyrosine 421 by displacing PTP1B from the invadopodium core [41]. In our work, we investigated whether PLXDC2 also regulates PTP1B to enhance the phosphorylation of Cortactin in GC cells. Our results showed that PLXDC2 was able to physically interact with PTP1B and prevent its dephosphorylating Cortactin at Y421. Therefore, PLXDC2 prevented PTP1B from dephosphorylating of p-Cortactin at Y421 by using a mechanism different from Mena^INV^.

In vitro, invadopodia are commonly identified as dot-shaped areas of degraded fluorescently labeled ECM proteins that colocalize with invadopodia-associated protein components, or as the spots of co-localization of F-actin and invadopodium markers after fluorescence staining. Although the invadopodia contain a variety of proteins, only Cortactin, Tks5 and MMP14 have been widely used as molecular markers of invadopodia so far. Cortactin, an important scaffold protein, acts as actin nucleation-promoting and actin branch–stabilizing factor during invadopodium formation, thereby being widely used as an invadopodium marker [42, 43]. Nevertheless, Cortactin is also enriched within lamellipodia and is used as a lamellipodia marker [44], making it essential but not a specific marker for invadopodia. Similar to Cortactin, c-Src substrate protein Tks5 is also a scaffold protein involved in invadopodium assembly [45] and serves as an invadopodium marker [25]. Tks5 harbors a Phox Homology (PX) domain, several proline-rich motifs (PRMs), and five SRC Homology 3 (SH3) domains[46]. With the SH3 domain, Tks5 interacts with N-WASP and c-Src-phosphorylated Tks5 interacts with Nck1, linking Tks5 to invadopodial F-actin assembly. Through the PX domain, Tks5 binds membrane phosphoinositides PI(3)P and PI(3,4)P2, thereby anchoring to the membrane [47]. However, overexpression of Tks5 in Tks4 null cells, which have incomplete podosome/invadopodia formation and inhibited ECM degradation, can only rescue their invadopodium formation but not localization of MMP14 for ECM degradation, suggesting that Tks5 can indicate invadopodium formation, but not its function [48]. Numerous studies have proven that invadopodia are the sites of surface MMP14 accumulation and MMP14 is the main invadopodial ECM protease, so MMP14 serves as a functional marker of invadopodia [42, 49]. In the present study, we selected MMP14 as invadopdium marker and using colocalization spots of MMP14 and F-actin under fluorescence staining to identify the invadopodium formation. Under these conditions, identified invadopodia should be functional and easy to be observed and quantified.

## Conclusion

In summary, the current study demonstrates that elevated PLXDC2 in GC is positively correlated with the Neoplasm Histological Grade, T Stage and N Stage as well as the poor prognosis of the patients. PLXDC2 enhances the level of p-Cortactin by physically interacting with PTP1B to promote the formation of invadopodia, thereby facilitating the invasion and metastasis of GC. PLXDC2 may serve as a new prognostic indicator and a potential therapeutic target for GC.

## Supplementary Information

Below is the link to the electronic supplementary material.

Supplementary file1 (PDF 882 kb)

Supplementary file2 (DOCX 15 kb)

## Data Availability

The datasets used and/or analyzed during the current study are available from the corresponding author on reasonable request.

## Abbreviations

GC: Gastric cancer
PLXDC2: Plexin-domain containing 2
TEM7: Tumor endothelial marker 7
TEMTR: TEM7-related
PEDF: Pigment epithelial-derived factor
MMP14: Matrix metalloproteinase 14
MT1-MMP: Membrane-type 1 matrix metalloproteinase
PTP1B: Protein tyrosine phosphatase 1B
ECM: Extracellular matrix
p-Cortactin: Phosphorylated Cortactin
