# Supplementary material for: PLXDC2 enhances invadopodium formation to promote invasion and metastasis of gastric cancer cells via interacting with PTP1B
Source: Clin Exp Metastasis. 2022 Jun 4;39(4):691–710. doi: 10.1007/s10585-022-10168-5 (PMC9338914; doi:10.1007/s10585-022-10168-5)
Supplement: Supplementary file 1 — Supplementary file1 (PDF 882 kb) [file 10585_2022_10168_MOESM1_ESM.pdf]

PLXDC2 enhances invadopodium formation to promote invasion and metastasis of gastric cancer cells via interacting with PTP1B

Bin Wu, *et al.*

Department of General Surgery and Center of Minimal Invasive Gastrointestinal Surgery, Southwest Hospital, Army Medical University (former Third Military Medical University), 400038 Chongqing, China.

Supplementary Tables

Table S1. Clinical features of patients with gastric cancer in our cohort.

Clinical Characteristic	Number	Percentage (%)
Age (years)		
<68[a]	93	54.71
≥68	77	45.29
Sex		
Male	119	70.00
Female	51	30.00
Histology grade		
G1	2	1.18
G2	66	38.82
G3	102	60.00
T stage		
T1	5	2.94
T2	15	8.82
T3	114	67.06
T4	36	21.18
N stage		
N0	45	26.47
N1	23	13.53
N2	43	25.29
N3	59	34.71
TNM stage		
I	12	7.06
II	55	32.35
III	103	60.59
IV	0	0.00
Tumor size (cm)		
≥ 5	117	68.82
< 5	53	31.18
Tumor site		
Proximal gastric	25	14.71
Middle gastric	58	34.12
Distal gastric	87	51.18

a Stomach cancer is most frequently diagnosed among people aged 65-74, and the median age at diagnosis is 68 (Surveillance Research Program (SRP) in NCI's Division of Cancer Control and Population Sciences (DCCPS).

Table S2. The information of GEO datasets used in this study

	GSE29272	GSE66229	GSE84433	GSE84437
Website	https://www.ncbi.nlm.nih.gov/geo/query/acc.cgi?acc=GSE29272	https://www.ncbi.nlm.nih.gov/geo/query/acc.cgi?acc=GSE66229	https://www.ncbi.nlm.nih.gov/geo/query/acc.cgi?acc=GSE84433	https://www.ncbi.nlm.nih.gov/geo/query/acc.cgi?acc=GSE84437
Organism	Homo sapiens	Homo sapiens	Homo sapiens	Homo sapiens
Country	USA	USA	South Korea	South Korea
Platform	GPL96	GPL570	GPL6947	GPL6947
Samples	268	400	357	433
Age, Sex, Stage, Grade	https://www.ncbi.nlm.nih.gov/geo/query/acc.cgi?acc=GSE29272	https://www.ncbi.nlm.nih.gov/geo/query/acc.cgi?acc=GSE66229	https://www.ncbi.nlm.nih.gov/geo/query/acc.cgi?acc=GSE84433	https://www.ncbi.nlm.nih.gov/geo/query/acc.cgi?acc=GSE84437

Table S3. Sequences of shRNAs targeting PLXDC2 and scramble (Mock) used in this study.

Gene		Sequence
sh-*PLXDC2*-1	Forward	5′-GATCCGCAGGAGTTATGTTACTTTGCTTCAAGAGAGCAAAGTAACATAACTCCTGCTTTTTG-3′
	Reverse	5′-AATTCAAAAAGCAGGAGTTATGTTACTTTGCTCTCTTGAAGCAAAGTAACATAACTCCTGCG-3′
sh-*PLXDC2*-2	Forward	5′-GATCCGGAGAAGTCGTACATCGAATGTTCAAGAGACATTCGATGTACGACTTCTCCTTTTTG-3′
	Reverse	5′-AATTCAAAAAGGAGAAGTCGTACATCGAATGTCTCTTGAACATTCGATGTACGACTTCTCCG-3′
Scramble	Forward	5′-GATCCGTTCTCCGAACGTGTCACGTTTCAAGAGAACGTGACACGTTCGGAGAACTTTTTG-3′
	Reverse	5′-AATTCAAAAAGTTCTCCGAACGTGTCACGTTCTCTTGAAACGTGACACGTTCGGAGAACG-3′

Table S4. The sequences of siRNA targeted Cortactin and PTP1B.

gene	sequences
CTTN	GGGAGAATGTCTTTCAAGA
PTP1B	TCAAGACACTGAAGTTAGA

Table S5. Sequences of primers used for qRT-PCR in this study.

Gene		Primers sequences
PLXDC2	Forward	5′-CCAGTTTCAGTTCGCCGATG-3′
	Reverse	5′-TGTCTACCGCCTTGAGAAAGT-3′
PTP1B	Forward	5′ -GACGAGGACCATGCACTGAG-3′
	Reverse	5′-GGAGGAGGGTCAGGCTATGT-3′
β-actin	Forward	5′-TTGCGTTACACCCTTTCTTG-3′
	Reverse	5′-CACCTTCACCGTTCCAGTTT-3′

Table S6. Primary antibodies used in this study.

Target	Dilution	Company	Catalog Number
PLXDC2	1:2000	Abcam	ab67226
Cortactin	1:1000	CST	3503S
p-Cortactin	1:1000	CST	4569S
PTP1B	1:2000	Proteintech	11334-1-AP
c-Myc	1:1000	CST	18583S
Flag	1:1000	CST	14793S
β-actin	1:5000	Abcam	ab8226

Supplementary Figures

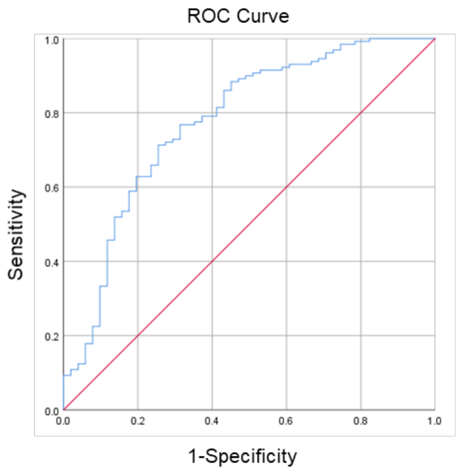

Area under the curve

Area	Std.Error	Asymptotic Sig	Asymptotic 95% Confidence Interval	
			Lower Bound	Upper Bound
.777	.401	.000	.697	.857

Cut off value	Sensitivity	Specificity
0.138	0.724	0.235

Figure S1. The ROC curve defines the cutoff value of PLXDC2 IHC scores for GC tissues in our cohort.

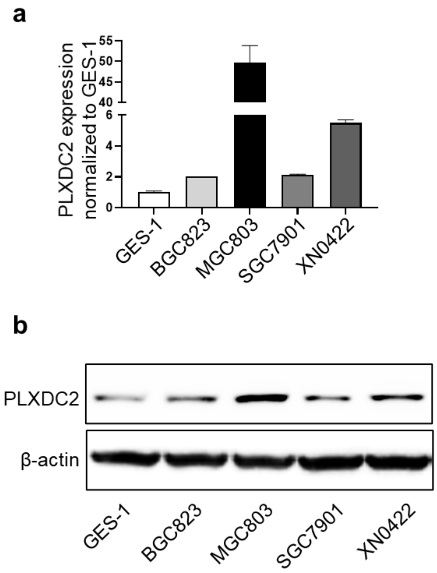

Figure S2. Expression levels of PLXDC2 in gastric epithelium and gastric cancer cell lines.

a. qRT-PCR assay showed that mRNA level of PLXDC2 expression in GC cell lines MGC803, BGC823, SGC7901 and XN0422 was higher than in immortalized gastric epithelium cell line (GES-1). **b.** Western blotting assay showed that PLXDC2 expression at protein level was higher in GC cell lines than in GES-1 cells.

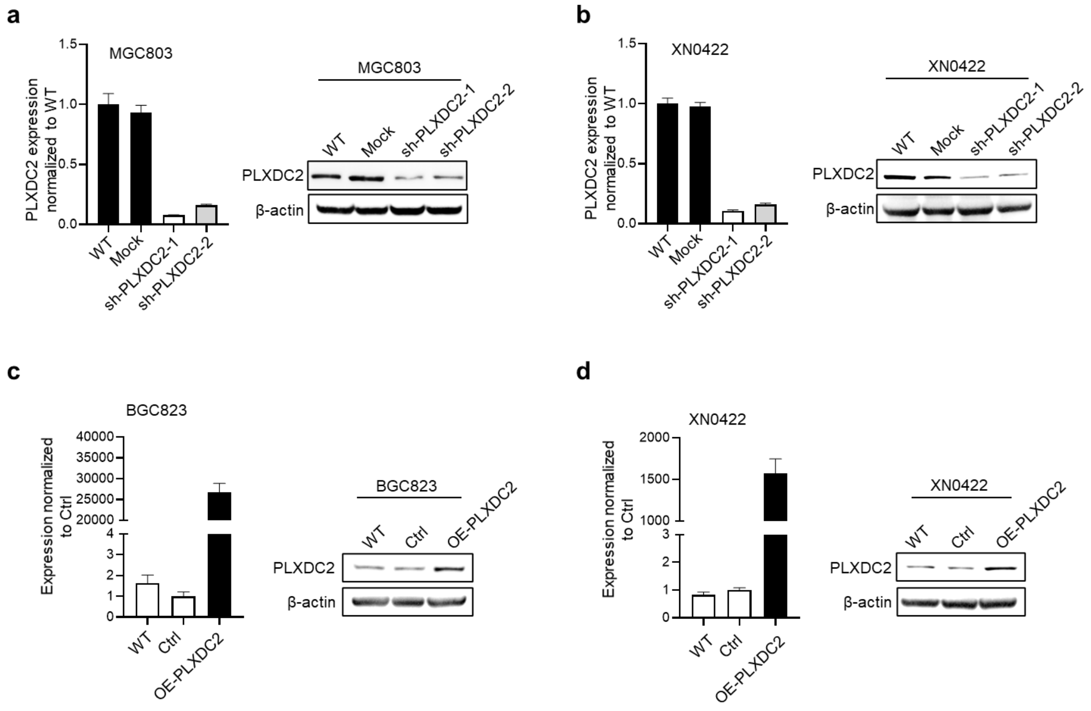

Figure S3. The efficiencies of PLXDC2 knockdown and -overexpression in gastric cancer cells.

a. The efficiency of PLXDC2 knockdown in MGC803 cells examined by qRT-PCR and Western blotting analyses. **b.** The efficiency of PLXDC2 knockdown in XN0422 cells examined by qRT-PCR and Western blotting analyses. **c.** The efficiency of PLXDC2 overexpression in BGC823 cells examined by qRT-PCR and Western blotting analyses. **d.** The efficiency of PLXDC2 overexpression in XN0422 cells examined by qRT-PCR and Western blotting analyses.

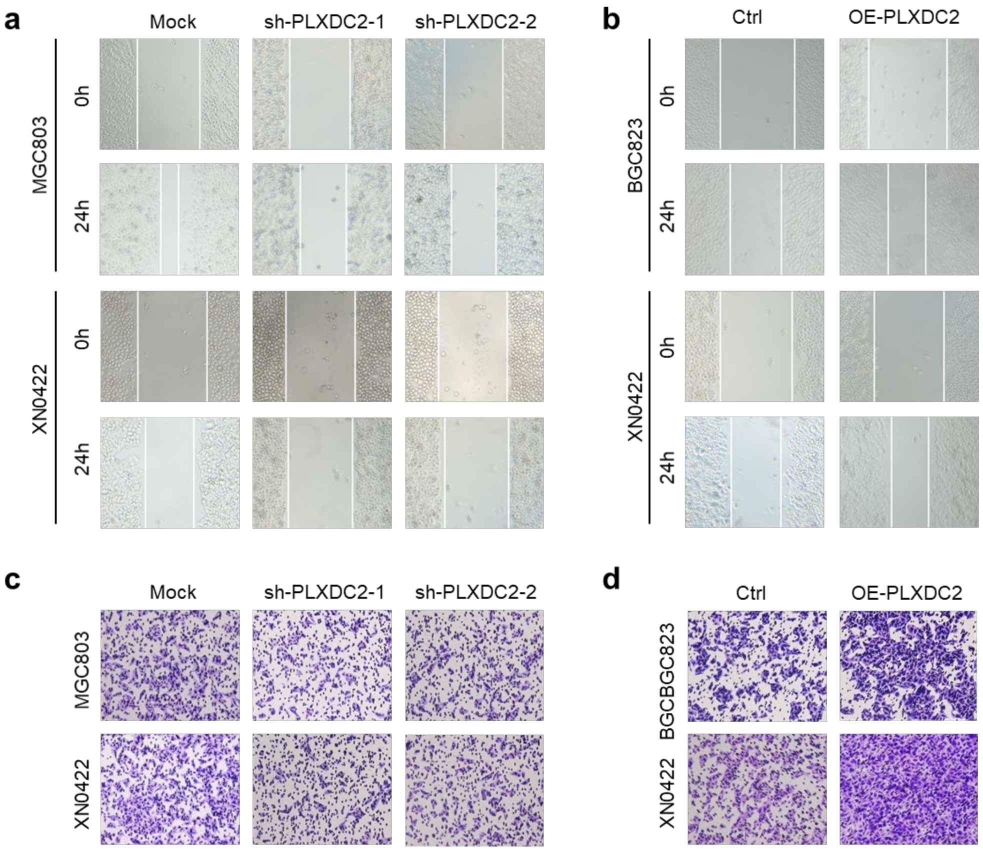

Figure S4. Representative images of the wound healing and transwell invasion assays for GC cells with PLXDC2-knockdown or -overexpression.

a. Representative images of wound healing assay for sh-PLXDC2 MGC803 and XN0422 cells and their Mock cells. **b.** Representative images of wound healing assay for OE-PLXDC2 BGC823 and XN0422 cells and their control (Ctrl) cells. **c.** Representative images of Matrigel-transwell invasion assay for PLXDC2-knockdown MGC803 and XN0422 cells and their Mock cells. **d.** Representative images of Matrigel-transwell invasion assay for OE-PLXDC2 BGC823 and XN0422 cells and their Ctrl cells.

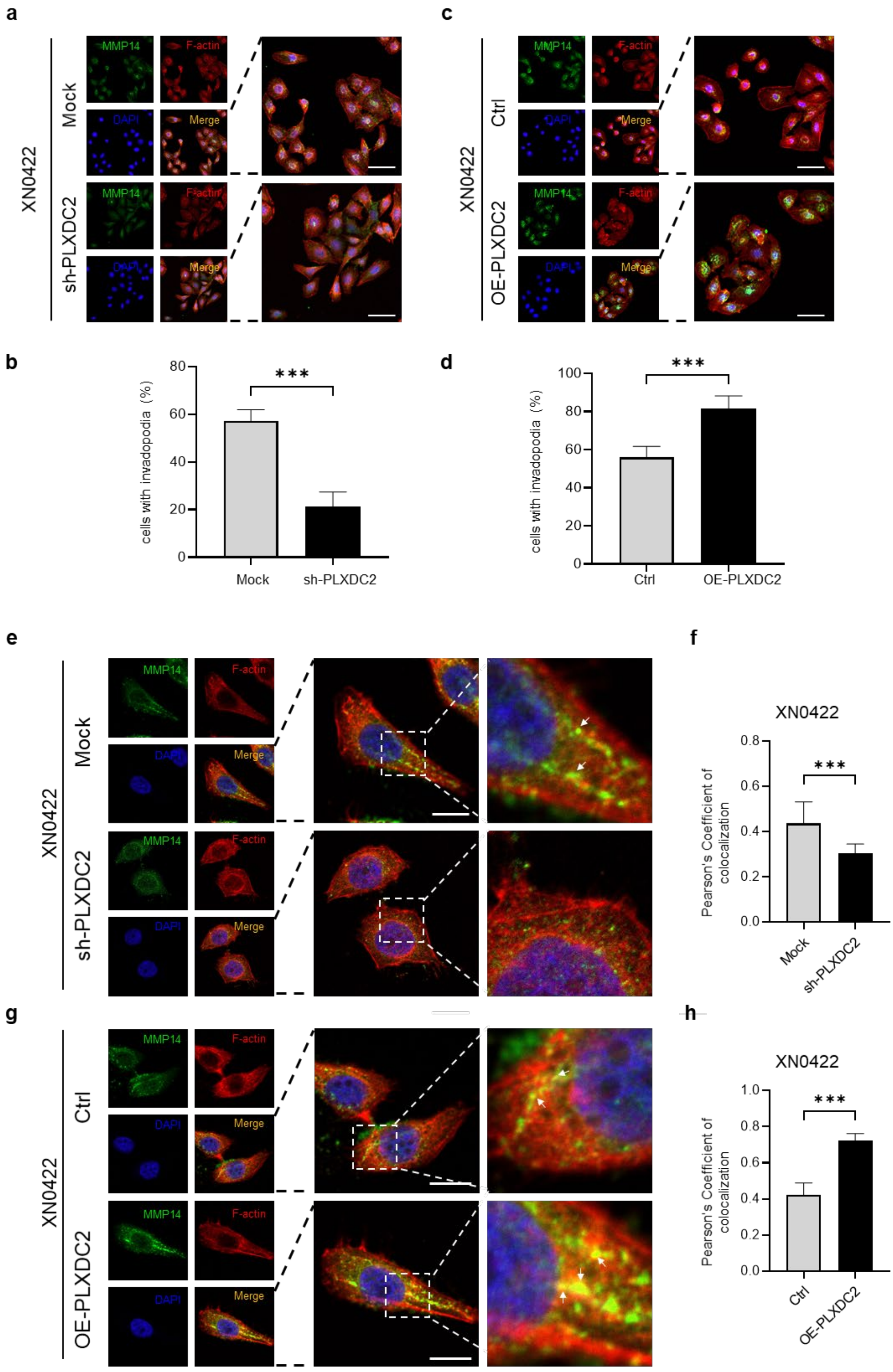
a
c
MMP14
F-actin
DAPI
Merge
XN0422
Mock
sh-PLXDC2
Ctrl
OE-PLXDC2
b
d
cells with invadopodia (%)
***
e
f
g
h
Pearson's Coefficient of colocalization
0.0
0.2
0.4
0.6
0.8
1.0
0
20
40
60
80
100

Figure S5. PLXDC2 is involved in invadopodium formation in XN0422 cells.

a. Representative IFC images showed that PLXDC2 knockdown decreased the proportion of invadopodia positive XN0422 cells. Invadopodia were defined as the co-localization (yellow spots) of MMP14 (green), an invadopodium marker, with F-actin (red). DAPI staining showed the Nuclei (blue). Scale bar = 40 μm. **b.** Statistical histogram showed reduced percentage of invadopodia positive cells in PLXDC2-knockdown XN0422 cells, as compared to Mock cells (5 random fields (10×), about 50 cells/field). Error bar, mean ± SEM, Student's t test. ***, $P < 0.001$. **c.** Representative IFC images showed that PLXDC2 overexpression increased proportion of invadopodia positive XN0422 cells. **d.** Statistical histogram showed that PLXDC2 overexpression increased the percentage of XN0422 cells with invadopodia. Error bar, mean ± SEM, Student's t test. ***, $P < 0.001$. **e.** Representative IFC images showed that PLXDC2 knockdown decreased the quantity of invadopodia in invadopodium positive XN0422 cells. White arrows indicate invadopodia. **f.** Statistical histogram showed that PLXDC2 knockdown decreased quantity of invadopodia in invadopodium positiveMGC803 cells, which was expressed as Pearson's coefficient of co-localization (MMP14 and F-actin) from 5 random fields (100×). Error bar, mean ± SEM, Student's t test. ***, $P < 0.001$. **g.** PLXDC2 overexpression increased quantity of invadopodia in invadopodium positive XN0422 cells. White arrows indicate invadopodia. **h.** Statistical histogram showed that PLXDC2 overexpression increased the quantity of invadopodia in invadopodium positive XN0422 cells (100×, 5 random fields). Error bar, mean ± SEM, Student's t

test. ***, $P < 0.001$.

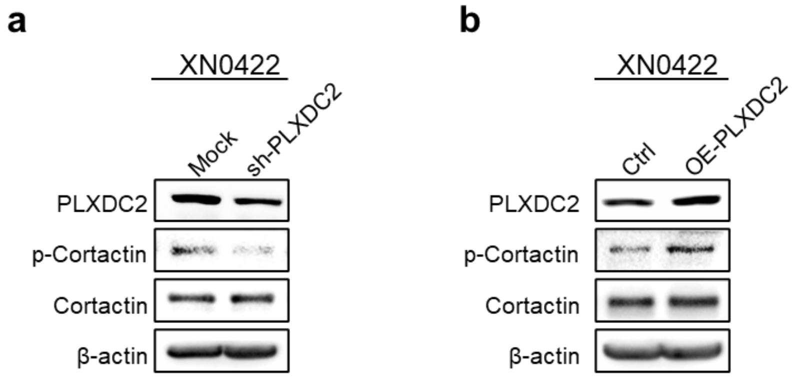

Figure S6. Manipulating PLXDC2 expression changes p-Cortactin level in XN0422 cells.

a. Western blotting analysis showed that PLXDC2 knockdown significantly reduced the level of p-Cortactin in XN0422 cells. **b.** Western blotting analysis showed that PLXDC2 overexpression significantly increased the level of p-Cortactin in XN0422 cells.

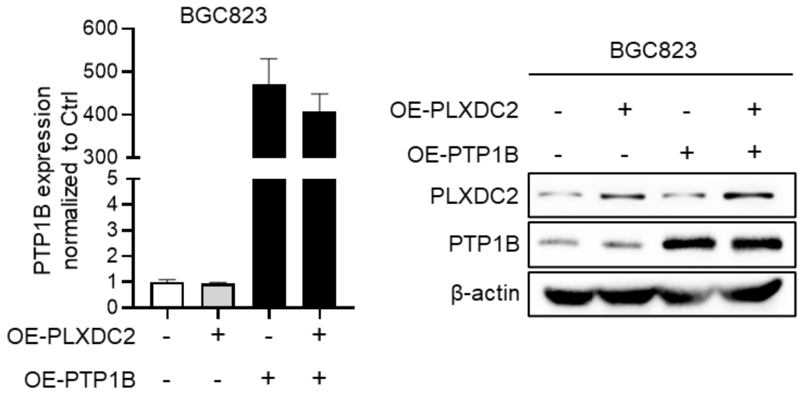

Figure S7.The efficiencies of PTP1B overexpression in OE-PLXDC2/Ctrl BGC823 cells.

The efficiencies of PTP1B overexpression in OE-PLXDC2 and Ctrl BGC823 cells

examined by qRT-PCR and Western blotting.
