# Supplementary material for: PLXDC2 enhances invadopodium formation to promote invasion and metastasis of gastric cancer cells via interacting with PTP1B
Source: Clin Exp Metastasis. 2022 Jun 4;39(4):691–710. doi: 10.1007/s10585-022-10168-5 (PMC9338914; doi:10.1007/s10585-022-10168-5)
Supplement: Supplementary file 2 — Supplementary file2 (DOCX 15 kb) [file 10585_2022_10168_MOESM2_ESM.docx]

## Supplementary Information

The datasets used and/or analyzed during the current study are available from the corresponding author on reasonable request.
